# Gender variations in access, choice to use and cleaning of shared latrines; experiences from Kampala Slums, Uganda

**DOI:** 10.1186/1471-2458-14-1180

**Published:** 2014-11-19

**Authors:** Japheth Kwiringira, Peter Atekyereza, Charles Niwagaba, Isabel Günther

**Affiliations:** Department of Sociology and Anthropology, School of Social Sciences, College of Humanities and Social Sciences; Makerere University, P.O. Box 7062, Kampala, Uganda; Department of Sociology, Kyambogo University, Kyambogo, P. O. Box 1, Kampala, Uganda; Department of Civil and Environmental Engineering, Makerere University, P. O. Box 7062, Kampala, Uganda; Swiss Federal Institute of Technology –Zürich (ETH-Z) and Centre for Development and Cooperation (NADEL), Zürich, Switzerland

**Keywords:** Gender, WASH, Latrines, Access, Cleaning, Slums, Kampala

## Abstract

**Background:**

Sanitation is one of the most intimate issues that affect women, especially in slums of developing countries. There are few studies that have paid attention to the gender variations in access, choice to use and cleaning of shared latrines in slums.

**Methods:**

This paper draws on qualitative data from a cross sectional study conducted between 2012 and 2013 in six slums of Kampala City, Uganda. The study involved both women and men. Data were collected from 12 Focus Group Discussions (FGDs), 15 Key informant interviews; community transects and photographs of shared latrines.

**Results:**

Location of a shared latrine facility, distance, filthy, narrow and irregular paths; the time when a facility is visited (day or night), privacy and steep inclines were gender ‘filters’ to accessing shared latrines. A full latrine pit was more likely to inhibit access to and choice of a facility for women than men. Results indicate that the available coping mechanisms turned out to be gendered, with fewer options available for women than men. On the whole, women sought for privacy, easy reach, self-respect and esteem, cleanliness and privacy than men. While men like women also wanted clean facilities for use; they (men) were not keen on cleaning these facilities. The cleaning of shared latrines was seen by both women and men as a role for women.

**Conclusion:**

The presence of sanitation facilities as the first step in the access, choice, use, and cleaning by both women and men has distinct motivations and limitations along gender lines. The study confirms that the use and cleaning of latrines is regulated by gender in daily living. Using a latrine for women was much more than relieving oneself: it involved security, intimacy and health concerns.

**Electronic supplementary material:**

The online version of this article (doi:10.1186/1471-2458-14-1180) contains supplementary material, which is available to authorized users.

## Background

Sanitation is one of the most intimate issues that affect women, especially in slums of developing countries. There are few studies that have paid attention to the gender variations in access, choice to use and cleaning of shared latrines in slums [1–4]. In low income urban settlements with few or no private sanitation facilities, women (those that can afford) have to queue for long periods to access public facilities (where they exist). As a coping mechanism, some women have to bear the embarrassment of having to defecate in the open, which exposes them to the possibility of sexual harassment or assault [4, 5]. Available literature on gender, women and sanitation does not analyse the intricate issues of access, choice to use and cleaning in developing countries, leave alone in urban poor settings or shared facilities [6–9]. This reality shows the magnitude of the sanitation challenge facing women. Due to their physical anatomy, values, upbringing and susceptibility to attack, women do not discreetly relieve themselves in open spaces. Consequently, women have no choice but to wait until dark, usually early in the morning when there is less risk of being waylaid or attacked for rape or assault [10–12]. Currently, there is little gender analysis and consideration in WASH interventions, development, monitoring and policy.

For a woman in a slum, going to ease herself is usually a daunting task; far beyond getting rid of excreta. The process often means squatting in a private spot or waking up before dawn to queue at public latrines [13–15]. For women in slums, a long wait at the public toilet means children are left unattended, or that a household chore is delayed. Unhygienic shared latrines threaten the health of women, by exposing them to reproductive tract infections [16–18]. For women who are menstruating, the need for adequate sanitation becomes even more acute [19]. Moreover, because women are generally responsible for the disposal of child waste in most households, when the provision of sanitation is lacking, women are more susceptible to diseases associated with human excreta.

Despite all this, the sanitation crisis affecting women has not been given the priority it deserves. United Nations and other international bodies tend to confine women’s issues to reproductive health and education [4, 20]. Although women’s distant access to water in both rural and urban areas and its health implications (including severe back pain caused by carrying heavy vessels of water over long distances) has been the subject of several studies, women’s lack of access to sanitation has not received the same attention. Even when the Millennium Development Goals (MDGs) and other global agencies (World Health Organisation, UNICEF and USAID) have been emphatic on the need for improved access to sanitation, few, if any governments focus on the impact of inadequate sanitation on urban poor women. Preliminary UN-Habitat analysis indicates the need to further study sanitation among women in slums, on the basis of increased health risks among women and children in particular [11, 21, 22].

The current status of latrines in the slums of Kampala reflects a glaring sanitation gap [23–27]. For instance; majority (70%) of the urban poor in Kampala use shared latrines and of these, less than half (47%) are clean enough to be used. Studies also indicate that another 45% of the shared facilities are abandoned within 5 years of commissioning [28, 29]. Women are more likely to be affected by the absence of latrines just like poorly constructed, poorly maintained, hard to access and those located far from the house. To-date, there is no known study presenting gendered findings on access, choice to use and cleaning of shared sanitation facilities in the slums of Kampala city.

## Methods

The study used a participatory cross-sectional design with qualitative methods for data collection. This paper mainly draws on qualitative data which facilitated an in-depth understanding of the gender variations in slum sanitation especially access, choice to use and cleaning of shared latrines. The study was conducted between 2012 and 2013 in six slums of Kampala City. The selected zones were: Dobbi, in Makerere –III Parish Kawempe Division; Kisenyi –I, in Kamwokya -II Parish Central Division; Gogonya –I, in Nsambya Parish Makindye Division; White Nile, in Kabalagala Parish Makindye Division and Jjuko, in Kibuye –I Parish Makindye Division. These selected slums exhibited low socio-economic indices especially; high average latrine user densities, low number of private latrines, many shared latrines, a high percentage of the latrine pits being full, low average incomes, big average household sizes, poor latrine cleanliness, low education levels among the populace, a high incidence of diarrhoea among children and low average asset indices [28]. After selecting the study zones, Google earth maps were used to divide each of the selected zones into two relatively equal parts, so as to have two different starting points for data collection. For each zone, two research assistants were assigned one starting point each. The purpose of these starting points and the use of up to-date maps were to minimize overlaps and exclusion due to lack of planning and congestion.

The study involved both women and men. Data were collected from 12 Focus Group Discussions (FGDs) with each focus group discussion homogenously composed with 8-10 participants. FGD participants were adult female or male residents in the study zone for more than five years who voluntarily offered to discuss sanitation issues openly with fellow residents. Once the participants had gathered, the moderator introduced the topic as a guide to the discussions [30–35]. In addition, 15 Key Informant Interviews (with landlords, local leaders and agencies that provided sanitation to slum dwellers) were conducted. Key informants were; persons involved in sanitation for the urban poor, property owners in study areas, public health providers (directly or indirectly) and leaders (technical or political) in the city. These key informants freely offered their time to be interviewed. Both women and men were taken through a participatory process to review their roles in provision and maintenance of shared latrines. Descriptive statistics on mode of facility construction, type of structure (improved or unimproved) and use modalities (free shared use, or pay per use) were collected from respondents. An observation checklist was used for the state of different sanitation facilities especially cleanliness, limitations of access, how full the latrine was and size of the latrine cubicle. In addition, pictures of shared latrines were taken. All images in this paper (refer to Additional file 1: Gender variations photo file) are original and no one has a claim on them whatsoever.

Community transects within the slums were done as a means to better appreciate the general sanitation situation in slums. Community transects involved a walk across the slums while taking notes and photographs per the transect checklist. The issues and evidence sought for and found were; poor drainage, foul smells from latrines and decomposing carcasses, uncollected garbage, open defecation, poor access to shared latrines and abandoned latrines.

The adopted analytical framework critiques the absence of gender responsiveness in sanitation that would address the unique challenges of women. The current conceptualization and design of latrines in slums is androcentric, androcentrism is the practice of consciously or otherwise making the male Worldview subsume female identity, culture and history [36–38]. Access is used to imply efforts and the process of *approach*(*ing*), *entering*, *exiting*, ‘*communicating with*’ (*interfacing with*), or making *use of* and to some degree, underlying *ease* and *suitability*. ‘Choice’ has been used as an act of *selecting* or *making a decision* when faced with two or more possibilities. ‘Cleaning’ is understood as the process of *enabling* and attempting to keep the available sanitation facilities *free from dirt*, *stain*, *or impurities*. The purpose of the paper is to show that there are underlying gender variations between men and women in access, choice to use and cleaning of shared latrines.

As a process, local (council) leaders helped the researchers identify venues for interviews. On average, key informant interviews lasted 45 minutes while FGDs took 60-90 minutes. The main issues for discussion were access to shared latrines, choice to use and cleaning by women and men. To address the likelihood of inhibition, female FGDs were conducted by female research assistants and those for males were conducted by male research assistants. In addition, training of research assistants on techniques of data collection such as probing helped to make FGDs an effective approach for data collection. When other community members showed interest in joining the FGDs after the maximum number of 10 people had been obtained, they were not allowed to join. They were told by the community leader to wait and members of the research team talked to them briefly about the purpose of the study. Verbatim quotations were identified and analysed, these have been used as part of the study findings.

### Quality assurance

For data collection, four graduate research assistants who knew most of the local languages (Luganda, English, Swahili, Lusoga and Runyakitara) in the slums of Kampala were recruited. In addition, these research assistants had experience in conducting focus group discussions and interviews. The research assistants were trained for two days and also participated in pre-testing the data collection tools. Daily field review meetings were held to clean the data and capture emerging issues for follow up and to provide guidance for further data collection. Both manual and electronic backups were used.

### Data management and analysis

Data were analysed manually using content thematic approach, following a framework advanced by Graneheim and Lundman [39, 40] to identify manifest and latent content in the discussion and interview scripts. Reports from FGDs and interview scripts were independently read several times to identify emerging themes and sub-themes. Joint discussions with research assistants were held to compare themes and sub-themes identified; a process that led to development of a unified list of codes for use in data analysis. The identified themes and subthemes were used to code data. Sub-group analysis was done, which involved examining the themes and sub-themes in relation to FGD data (men, women) and key informants. The data coding process began during data collection and went on until after data collection. This enhanced continuous analysis while also serving as an analytic method for coding and analysis [41]. Raw data consisting of interview transcripts, participant observation, field notes and photographs were coded. In first cycle coding, data were bigger in magnitude with the coding outcome ranging from a single word to a full sentence and sometimes to a set of sentences covering an entire page. In first round coding, data on access, choice and cleaning were separated in preparation for the second round of coding. In second cycle coding, the portions coded were based on the first cycle codes of access, choice and cleaning. The coding process proved heuristic and served as an exploratory technique to the gender variations in sanitation. Because of this systematic approach, the coding was not just labeling, it enabled the initial understanding of the sanitation problems in Kampala slums. Subsequently this process became sort of cyclic; from the data to the idea, and from the idea to all the data pertaining to gender variations in access, choice and cleaning of shared latrines. This served the purpose of compressing the analytical framework further based on patterns and filters [33].

### Ethical considerations

The study protocol was approved by the Research Committee for the School of Social Sciences, College of Humanities and Social Sciences Makerere University. This committee considered all technical and ethical issues of the study. Clearance was also obtained from local leaders in the respective slum zones. An introductory letter issued by Makerere University was presented to local leaders in addition to explaining the purpose of the study, confidentiality, voluntary participation; anonymity and freedom to withdrawal from the study were clearly explained [42]. Verbal consent to participate in the study was obtained from all study participants. Participants were free to withdraw from the study if they felt uncomfortable. No persons lacking capacity to consent were enrolled or involved for the study. In addition, study participants’ identifiers are not presented. The need for confidentiality was emphasized during training of research assistants prior to conducting of the study [43]. With the study findings being published, this shall reduce further resource wastage, for instance; by not conducting other studies in the same area without first benefiting from these findings. Publishing and information sharing minimizes community research fatigue and wastage of valuable resources [44, 45].

## Results

**Figure 1:**
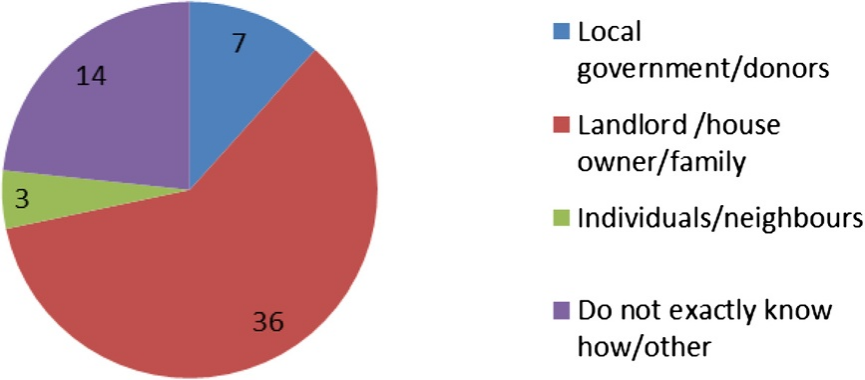
**Latrine ownership in the study zones.**

The study found that, there were numerous and sometimes ‘mixed’ ownership types originating from the nature of construction, land ownership on which the latrine facility was built and sometimes the initial arrangement prior to the facility being erected. For instance, a landlord who donated land for the construction of a community latrine later usurped this facility or dictated its terms of access and use. The various means of construction subsequently complicated access and use of these facilities with some previously communal or shared facilities reverting to private use after commissioning and in some cases, being altogether abandoned due to mismanagement.Findings show that 11% of the sanitation facilities were pay per use and therefore semi-public. Private, facilities were much fewer and were not the focus of the study as they are usually clean given the restricted access and few users (usually one household). Majority (81%) of the respondents used shared latrines, with most (89%) of them being un-improved. The rest of the facilities (about 8%) were improved (most of these improved facilities had been built by institutions such as government and donor agencies). Figure 1 shows the various ownership types of latrine facilities in the study zones.

### Access to a shared sanitation facility

The main themes identified and coded were; open defection, time of day, sexual harassment, affordability and cost of sanitation, alternative methods of excreta disposal, cleanliness, easy access, self-respect, catching infections, self-esteem and privacy. Verbatim quotations were identified and are part of the study findings. The study found that, access to a sanitation facility affects the use of a facility and its surroundings. When a latrine could not be easily accessed, the surroundings were usually littered with human waste.

Open defecation:

“When you find the latrine locked, you quickly go behind it and ease yourself before someone finds you…” Male FGD participant Jjuko Zone

Accessibility challenges:

“It is not appealing to climb a strip of stairs near children who are playing before you can access a latrine. On many occasions, these children have seen our underwear which they talk about the whole day. ..because of this, I only use the latrine when the children are not near or when I have no alternatives. For us elderly women, that latrine is not the first choice when we are in need of relieving ourselves.” Elderly Female FGD participant, Kisenyi I Zone

Photo 1 shows a typical slum location in Kampala city.

### Photo 1

Even when a facility may be near, clean and private but challenging to enter and use, it serves no utility to some groups especially women and girls. The mode of accessing a facility such as the one in photo 2 renders a facility unattractive and unsafe to many females.

### Photo 2

Time of day and sexual harassment:

“After dark, these latrines are mainly used by men. Women fear going out especially when it is raining, because, even if you shouted when under attack, no one will hear you. Here when it is dark and raining women do not use latrines”. Local leader, White Nile zone

Access to a sanitation facility is the first interface point and is crucial in providing sustainable sanitation. The other accessibility themes identified were; few latrines in slums that were not easily accessible. Accessibility challenges related to filth on the way, narrow paths and steep inclines that made access a challenge especially to female users. In as far as conceptualizing sanitation is concerned, findings show few areas of convergence between women and men. For instance, while many women mentioned access to latrines being constrained during the night because latrines are far and locked; in comparison, men generally did not experience such a limitation. This finding is consistent with other studies conducted in the developing world [13, 46–48] . In coping with the problem of accessing latrines that are far at night, women had to find someone to company them in view of the possible dangers of assault or rape in the night. Although latrines were not far in the sense of long distances, they were not located within the yard of the dwelling unit. What made this distance unappealing was where one had to pass; through back alleys and meandering in a congested neighbourhood rife with filth. The distance being discussed is not more than 50 metres on average. It is such realities that discourage women from accessing the few available sanitation facilities. Photo 3 shows the gendered challenges in a bid to access a sanitation facility.

### Photo 3

The reported modes of coping in an attempt to mitigate the above challenges include using buckets or plastic bags as makeshift toilets inside their shelters. This finding is in agreement with Feachem et al [17]. Pictorial evidence in photo 4 is typical of the embedded gendered strategies in coping with the sanitation challenge in the slums of Kampala. The potty on the roof of the makeshift dwelling unit shown in photo 4 had a dual utility; it was officially and conveniently a waste management accessory for the ‘baby’ even when there was no baby in the household.

### Photo 4

The potty was one of the highly regarded household devices since it was used by the stay home female adults in substitution for a latrine to save money. In slums, clean latrines were ‘pay per use’ facilities costing between US$ 0.12 –US$ 0.2 per use, therefore expensive for the non- working adults, especially females. This made such facilities unaffordable. On the other hand, most of the shared latrines were free but filthy. This finding is consistent with Rutstein [49].

Lack of affordability:

“It is not possible to pay for a latrine before I find food for my children. Unless I am sure about what to eat; I cannot pay to defecate. For us here we live from meal to meal. That is why I do not use pay per use latrines even when I know that they are very clean.” Female FGD participant White Nile zone

**Figure 2:**
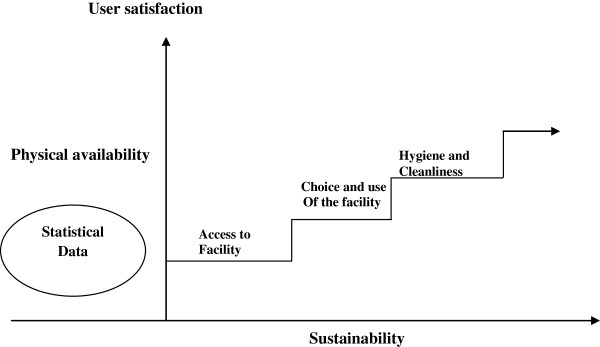
**The sanitation utilization ladder.**

Findings indicate that sanitation access, expectations and roles were varied along gender lines, with some roles being perfomed by both women and men while other roles; especially cleaning seen as specifically a women’ role. Overall, women preferred privacy and convienence more than men. Table 1 shows the varied gender roles in access to a sanitation facility with a presentation of general enablers and constraints.Beyond the physical availability of sanitation facilities, is access to a given facility; by time of day, by age, physical abilities and gender. The mode of access to a sanitation facility then determines choice and use against the ease or difficulty embedded by each prospective user category, but more specifically for women and men. Choice and use of a given sanitation facility is then sustained by the hygiene and cleanliness levels of the facility in question, which altogether increase user satisfaction. Study findings showed that generally, women had less access capabilities than men. It is this continuum presented in Figure 2 that constitutes the sanitation utilization ladder.

**Table 1:** **Gender variations in access to shared latrines**

Male		Female	
Positive aspects	Negative aspects	Positive aspects	Negative aspects
Access			
• Can provide resources and labour to construct a latrines which improves access	• Generally not interested in covert things like sanitation access	• Can easily talk to neighbors and friends about sanitation and lobby to increase access	• Women tend to have low incomes with numerous needs and therefore may not have what to spend on sanitation access
• Tend to have better incomes that can be used for sanitation access	• When under the influence of alcohol, they do not mind using a latrine or not. This makes latrine access a secondary concern	• Tend to pay detailed attention to household hygiene which works towards improved sanitation access	• Women not eager to access poorly located and distant latrine facilities
• Time of day may not determine accessing a sanitation facility	• Can easily access poorly located and poorly lit and risky facilities		• Women not eager to find latrines at night
• Filth along the way to the sanitation facility may not easily hinder males from accessing a latrine			• A filthy path may cause one to post pone the decision to ease oneself or cope with open defecation.
• Distance is not a crucial variable in access to a sanitation facility			• Distance can impede access to a sanitation facility
• Men usually not at home and have better options of sanitation facility access			• Fear of being attacked and harmed at night
			• Usually at home and have limited choice on which facility to use

### Choice to use a shared sanitation facility

One of the key themes in this study was respondent considerations on choice of a sanitation facility and how this choice varied between women and men. Findings indicate that women were more inclined to a facility that was close to the dwelling unit, accessible during day and night time, a facility with cubicles big enough for entering with a child as well as for changing menstrual materials, a facility with adequate lighting and one that was lockable. Although a latrine with a lock was seen as secure, women were skeptical and suspicious about being locked in the latrine by malicious people. Some women reported that they would rather use a polythene bag or bucket than risk being locked inside a latrine facility especially at night. This is similar to findings of other studies [50, 51]. Women also feared that they would contract an illness due to the lack of cleanliness in the shared latrines. Interestingly, women rarely mentioned illnesses usually associated with poor sanitation such as diarrheal diseases. Instead, they (women), feared what they perceived to be the risk of catching a sexually transmitted infection (STI) from a dirty sanitation facility, the kind of illness which could raise the suspicion of a spouse or sexual partner. Some latrines were doorless (see photo 5), this posed varying challenges especially among females (women and girls) than males.

### Photo 5

Such doorless facilities were usually used by men at night which was not the case with women. This aspiration has a resource implication that makes sanitation a wider issue than previously thought.

One female FGD participant in Kisenyi rejoined;

“When the latrine is far from your home by the time you get there it is too late. At least for a man; he can just go anywhere and urinate so long as he turns his back on people; this is not the case for us women. We have to walk until we find a latrine. That is why we prefer to use basins at home”.

In order to understand the gender variations in choice of latrines, women and men were taken through a participatory process where each gender’ concerns were discussed. Table 2 shows the analysis of these variations along enablers and constraints of choosing a given sanitation facility.

**Table 2:** **Gender variations in choice to use shared latrines**

Male		Female	
Positive aspects	Negative aspects	Positive aspects	Negative aspects
Choice to use			
• Have power which they can invoke for latrine use	• Generally less bound to use latrines for urinating	• Most affected by poor hygiene: the negative motivation can cause positive results.	• May not initially train their children to use latrines properly due to culture and ignorance
• Do not mind sharing latrines with women	• Less at home and do not see latrine choice and access as a problem for family members	• Mostly want a safe facility for their children	
		• Want maximum privacy in the latrine	
	• Men can spend the little money they have to impress than on sanitation	• Want adequate space when teaching children how to use the latrine	
	• Can use door less and makeshift structures to relieve themselves	• Irrespective of religious concerns, women want water for adequate washing especially when menstruating	
			• With little money, women would rather buy water and food than spend it on sanitation
			• Do not want to share latrines with men

A female FGD participant in Gogonya asserted that;

“A woman cannot feel safe walking to the latrine at night. Men can rape you. Even during the day, you have to be careful given the narrow and slippery paths –you can easily fall in the dirty water”.

Choice of a facility, self-respect and privacy:

“You cannot use a sanitation facility where people outside hear and see you while you defecate. At least when you use a container in your house, you are alone. This type of latrine can only be used by young children and not an adult”. Female tenant Kisaasizi zone.

Hygienic latrines were mostly accessible on a ‘pay per use’ basis which turned out to be unaffordable to the majority of the women.

Lack of cleanliness and fear of catching infections:

“Our latrine lacks a cleaner and cleaning material, so when someone misuses the facility while in her periods and does not clean it afterwards, it becomes unusable. To avoid getting an infection, when I am in my period, I choose not to use the latrine”. Land Lady, Gogonya.

### Gender variations in cleaning shared sanitation facilities

Sanitation generally refers to the safe separation and disposal of urine and feaces, and also the management of garbage and wastewater [37]. The adequacy of sanitation facilities is often evaluated solely based on the extent of containing the waste and how the user is free from contamination. This section undertakes a gender understanding of cleaning the available shared sanitation facilities.

Focus Group Discussions with women and men as well as key informant interviews indicated gender variations in shared latrine cleaning. The analysis shows that females had more inclinations to keep latrines clean than men. Women had more cleaning roles and responsibilities than men, and not necessarily capabilities. This serves to show the overarching male World view as subsuming female needs, concerns and roles. See Table 3 for a detailed gender disaggregated variation of shared latrine cleaning between women and men.

**Table 3:** **Gender variations in shared latrine cleaning**

Male		Female	
Positive aspects	Negative aspects	Positive aspects	Negative aspects
• Men more than women can be co-opted in maintenance that is beyond cleaning	• Limited role in child care which is primary household sanitation and hygiene	• Mainly take on child care which involves a lot of toilet training	• Lack resources for cleaning and maintenance
• Willing to clean shared latrines for payment	• Cleaning of latrines and hygiene culturally seen as female roles	• Want to show their men that they are clean	
	• Think they are meant to deal with ‘out of home’ and ‘hard affairs’ and not the ‘soft’ aspects like sanitation	• Society thinks that women should mind domestic sanitation problems	
	• Peer pressure is for outward standards than private standards such as household hygiene	• Tend to be more mindful of latrine status.	
		•Peer pressure to sustain some hygiene standards works to keep latrines clean	
		• Women most likely to discuss hygiene and sanitation since they stay home (social groups among women)	

Men needed less privacy, and usually only needed a latrine to defecate, while women required a latrine both to urinate and to defecate. This situation was also found in previous studies that indicate the secrecy and stigma of sanitation among women [52, 53]. These gender variations meant that, men needed the use of a toilet less and therefore were not faced as often; with the problem of high latrine user fees or with the grim prospect of resorting to a dirty shared latrine. Findings also showed that, men were more likely to leave the community for work during the day, and thus potentially have access to more and better sanitation facilities at the place of work or elsewhere. Because of this, the status of shared sanitation facilities at home did not usually concern men. On the other hand, women found themselves in a sapping position with no clean sanitation facilities to use. Although the practices surrounding sanitation and hygiene are largely debilitating to women, there is a wide spectrum of different practices and taboos that vary from region to region, from culture to culture and country to country in different contexts [54].

Anatomically female and male differences cause more barriers for women compared to men. Despite the differences between women and men, the implications of these differences are many times overlooked and gender considerations sacrificed for convenience in construction and cost saving [16, 21, 37].

## Discussion

Improved sanitation in slums was conceptualized as incremental and dynamic. The first and inevitable step is the availability of sanitation facilities (in varying forms; improved or unimproved, onsite or off-site). This first step in sanitation delivers benchmarks and statistical data. Beyond the count and statistics, the more complex issues of user interface and maintenance come into play; affecting use, function and sustainability of a given facility. Access to a sanitation facility is crucial for the entire sanitation chain but has wide variations between females and males [12, 35, 55]. This variation is partly due to the female anatomy, physiology, socio-cultural expectations and roles [36, 56]. The need for gender disaggregated data in social service delivery is crucial and needs more emphasis when dealing with sanitation and other social determinants of health. The study has shown that, merely counting the number and type of sanitation facilities (improved or not) without minding the condition of these facilities and how they can be accessed by all potential users does not provide a complete picture. This proposed approach of going beyond numbers and mere structures enables an analysis of the unique cases that urban poor women and girls encounter [38, 57].

Once sanitation facilities cannot be accessed, they cannot be utilized. The most important issue after sanitation facilities have been provided is to ensure that they are accessible and usable. This is the first rung on the sanitation utilization ladder as shown in Figure 2, irrespective of whether the sanitation facility is improved or not. This is the potency of this paper beyond the traditional sanitation ladder. Once people gain access to sanitation facilities, they can then have a choice over which ones to use or not, depending on their cleanliness and hygiene levels. Tilley et al [12] argue that a gender-responsive approach to sanitation is a means of speaking to the needs and inclinations of a diversity of users. Therefore, a gender analysis of access to sanitation facilities addresses issues previously undiscussed including menstrual hygiene management and privacy which have the potential to make sanitation facilities widely used, maintained and sustainable [58–60]. The study also shows that, choice and use of an accessible sanitation facility shapes the hygiene and cleaning outcomes that in turn shape the wider maintenance agenda.

Irrespective of one’s religious affiliation, the presence of water was a great need by women to wash up. Women argued that sanitation facilities should not expose them to incidences of vaginal infections, and therefore should not be (almost) full. In addition, women did not want to be seen going to the latrine, probably due to the unhygienic nature of the shared latrines. Studies recognise that the needs and interests of women and men in sanitation differ considerably, and that these interests have important implications for the performance of the sector and beyond [12, 61]. Although many women did not admit to it, literature [19] and in-depth interviews pointed to the use of old clothes as menstrual pads because the disposable sanitary napkins were too expensive. This is in agreement with studies showing that an overwhelming majority (97%) of women resort to re-usable materials especially clothes for menstrual hygiene [12, 62]. Therefore, in the absence of disposable products, women attempt to stay clean with reusable sanitary materials by cleaning themselves while inside sanitation facilities that are not well equipped for this very private process. This gender dimension of latrine access and use is denied women when latrines are shared, unclean, small and not private enough for specific gender needs. This is more acute in slums than elsewhere.

Obtaining access to a sanitation facility alone is not enough, but only a crucial first step towards the embedded sustainability [63]. The user satisfaction -sustainability continuum (see Figure 2) includes other variables especially the range of the available choices for different uses and users. For instance, urinating or defecation (for women and men) and therefore, the extent to which gender concerns are addressed, cleanliness of the facility, number of users and type of the facility are crucial indices [27]. Studies have shown that the presence of sanitation facilities that are not clean has little or no impact in the prevention of sanitation-related diseases, especially for children [47, 64, 65]. Some authors have advocated for an inclusive nomenclature in sanitation in order to ascertain that both female and male concerns are addressed [66, 67].

The study did not explore post defecation hand washing as a measure of personal hygiene, although this is closely related to personal defecation practices. These findings should as well be understood as limited by their qualitative nature which represents sanitation in Kampala slums and not Kampala city. However, it serves good to remember that, not everything that counts is countable. Even then, critical sanitation insights can be credibly drawn from this paper for policy and planning given that 60% of Kampala dwellers reside in informal settlements and slums.

## Conclusion

There are inherent, but distinct gendered motivations and limitations in shared latrine access, choice to use and cleaning. This implies that, latrines need to accommodate both male and female uniqueness beyond the convenience to the builder and the financial implications. This knowledge calls for the origination of sanitation approaches in the design and implementation that are gender responsive. Among the urban poor, shared latrines for men and women need to be purged to enable women adequate privacy. If latrine use is to increase and be sustainable, then all shared sanitation facilities must be kept clean, private and functional. The understanding of the gendered interpretations of embarrassment, self-respect and safety need to feed into a greater understanding of shared latrine access, choice, use and cleaning for sustainable sanitation among the urban poor.

## Authors’ information

JK is a PhD Candidate at Makerere University and Lecturer of Sociology at Kyambogo University, Kampala Uganda; PA is Professor of Sociology at Makerere University, Kampala, Uganda. IG is Assistant Professor and Chair of Development Economics at Centre for Development and Cooperation at The Swiss Federal Institute of Technology –Zurich, Switzerland; CN is Senior Lecturer and Sanitation environmental Engineer at Makerere University in the College of Engineering Art and Design, Kampala Uganda.

## Electronic supplementary material

Additional file 1: **Gender variations photo file.** (DOCX 1 MB)

## Abbreviations

KCCA: Kampala Capital City Authority
LC: Local Council
FGDs: Focus Group Discussions
KII: Key Informant Interview
IDI: In Depth Interview
UN: United Nations
UTI: Urinary Tract Infections
ETH: Swiss Federal Institute of Technology
EAWAG: Department of water and Sanitation in Developing Countries
NADEL: Centre for Development and Cooperation
SANDEC: Swiss Aquatic Institute
VIP: Ventilated Improved Pit Latrine
MGDs: Millennium Development Goals.
