# Supplementary material for: Gender variations in access, choice to use and cleaning of shared latrines; experiences from Kampala Slums, Uganda
Source: BMC Public Health. 2014 Nov 19;14:1180. doi: 10.1186/1471-2458-14-1180 (PMC4247598; doi:10.1186/1471-2458-14-1180)

**Photo 1: A typical slum location in Kampala City**


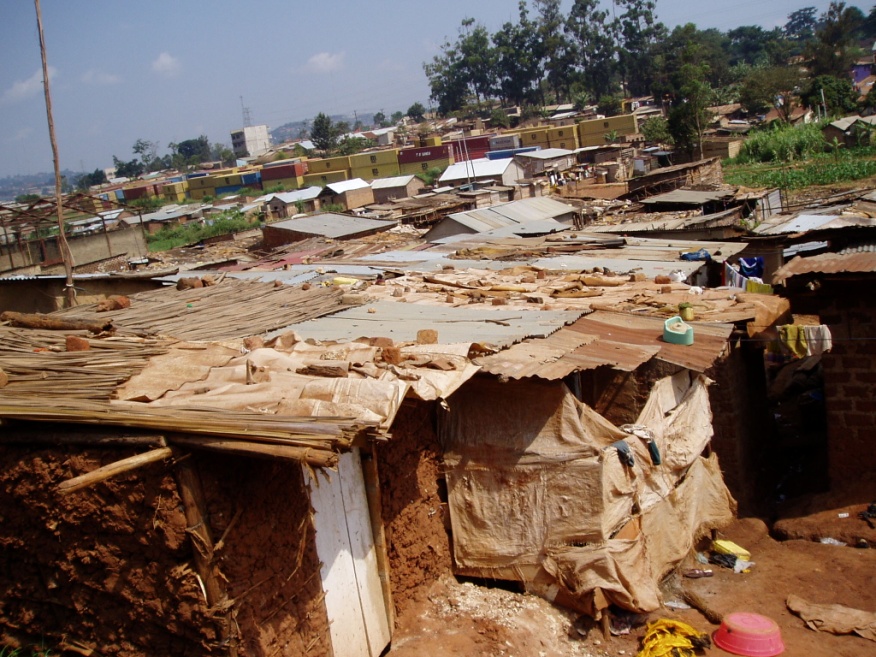


**Photo 2: A case of challenging access to latrine by Women**


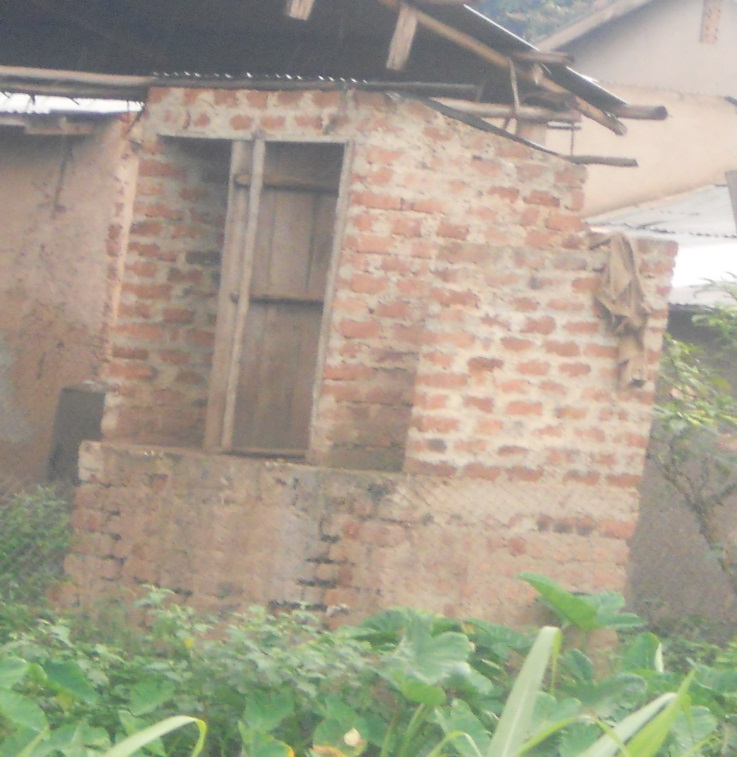


**Photo 3: A case of a discouraging route for a female latrine user**


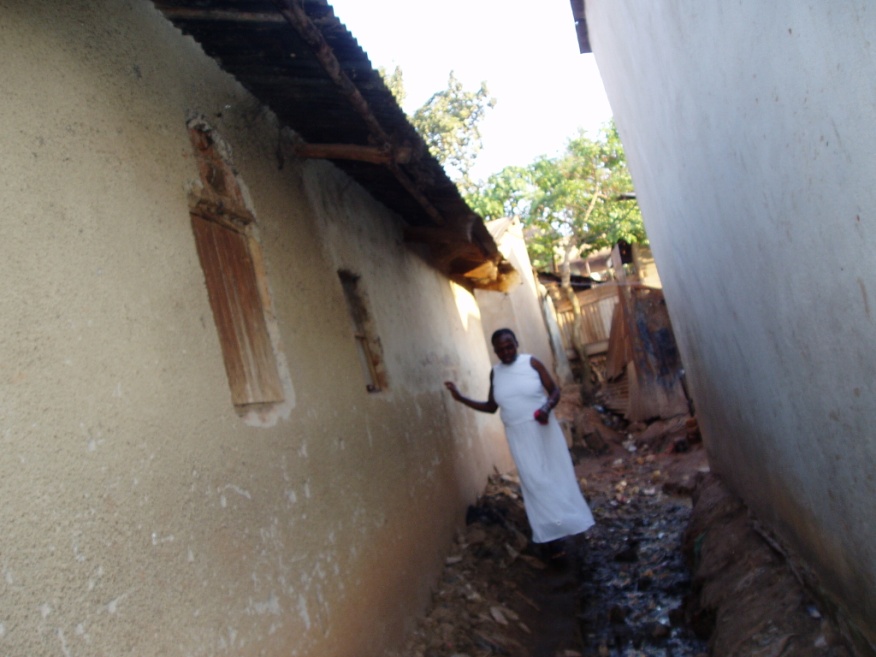


**Photo 4: A child potty for Human waste disposal among female adults**


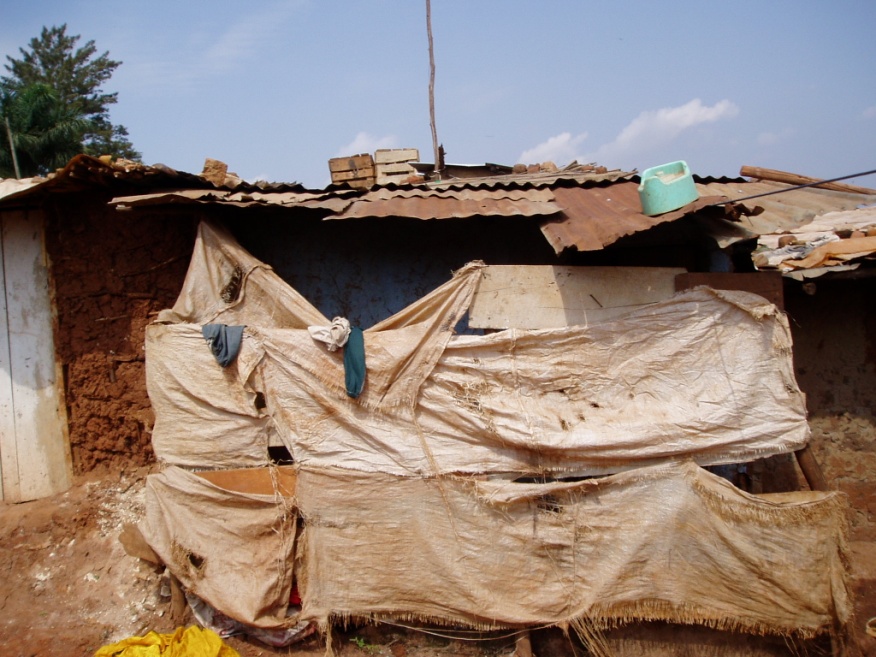


**Photo 5: Doorless latrines serve no purpose especailly for adult females**


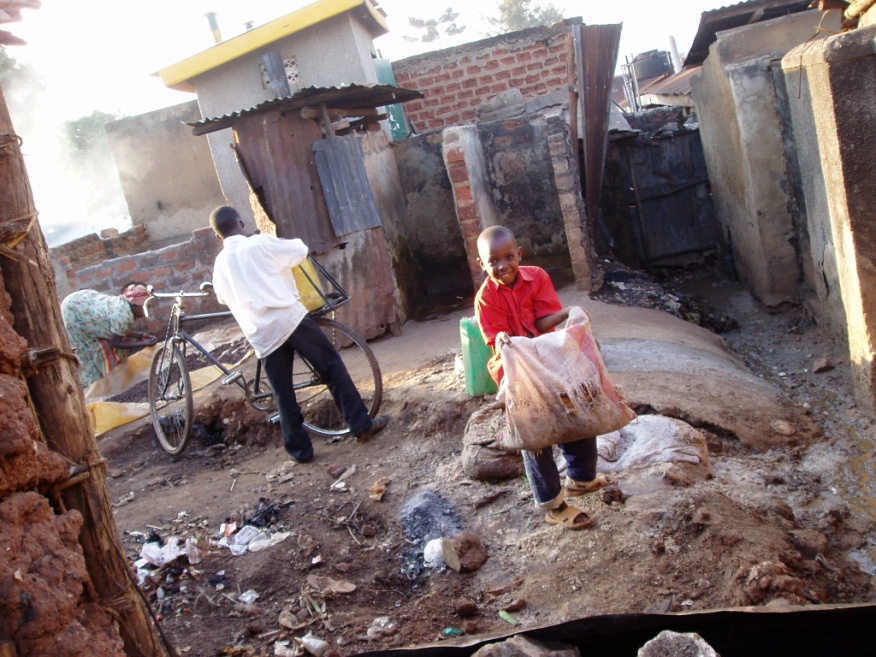

Supplement: Supplementary file 1 — Additional file 1: Gender variations photo file. (DOCX 1 MB) [file 12889_2014_7310_MOESM1_ESM.docx]
